# Using a hospital passport from the perspective of adults with intellectual disabilities, family carers and health professionals: A qualitative study

**DOI:** 10.1177/17446295221145996

**Published:** 2022-12-12

**Authors:** Freda McCormick, Lynne Marsh, Laurence Taggart, Michael Brown

**Affiliations:** School of Nursing and Midwifery, 1596 Queen’s University Belfast, Belfast, UK; School of Nursing and Midwifery, 1596 Queen’s University Belfast, Belfast, UK; Ulster University, Jordanstown, UK; School of Nursing and Midwifery, 1596 Queen’s University Belfast, Belfast, UK

**Keywords:** adult, family, health communication tool, intellectual disability, person-centred care, regional hospital passport

## Abstract

This article explores the experiences of the use of the *Regional Health and Social Care Hospital Passport (Regional Hospital Passport)* in Northern Ireland from the perspectives of adults with intellectual disabilities, family carers and health professionals. From semi-structured interviews three themes emerged: usefulness; facilitators; and barriers to the use of the *Regional Hospital Passport*. There were clear benefits of the Hospital Passport when used across hospital services and clinical practice settings such as dental and General Practices. There was participant agreement that communication and the person-centred care experiences were enhanced. Findings suggest that providers of health and social care services need to take greater responsibility and accountability for ensuring *Regional Hospital Passports* are promoted and used across all settings. There is a requirement to develop the wider use and uptake of hospital passports to support adults with intellectual disabilities, with potential for use with other patient groups.

## Introduction

Research findings suggest that approximately 2% of the world population have an intellectual disability (Public Health England, 2016) and it is anticipated that their health needs will increase due to a greater increase in life expectancy resulting in an ageing population (World Health Organisation, 2016). Consequently, people with intellectual disabilities across the lifespan are more vulnerable to experiencing complex physical and mental ill-health resulting in more frequent admissions and longer stays in acute hospital service when compared to the non-disabled (RQIA, 2018). Yet, adults with intellectual disabilities and their families continue to report dissatisfaction with their care when accessing primary care and acute general hospital services (Learning Disabilities Mortality Review (LeDeR) Programme, 2018; MENCAP, 2007) warranting further attention.

There is growing evidence of the barriers to the provision of healthcare for adults with intellectual disabilities relating to communication problems and poor sharing of health information (Howieson, 2015; Mastebroek, et al., 2014; Walker, et al., 2014). Often, health professionals in acute care settings are unaware that a patient has an intellectual disability until admission (Tuffrey-Wijne et al., 2014). Therefore, they may be poorly prepared to provide safe, effective, and appropriate care and support. This is problematic as health and communication information is critical to inform the plan of care within the acute hospital setting. Therefore, they need to understand the increasing complexities of patients with cognitive and communication needs such as children and adults with intellectual disabilities and autistic spectrum disorder with the need to make reasonable adjustments to their care and support where necessary (Tuffrey-Wijne et al., 2014). The *Health and Social Care Hospital Passport* is a regional Northern Ireland tool that has been utilised by adults with intellectual disabilities, their families and health professionals to enable safe and effective person-centred care and throughout the care journey.

A mixed methods study in Canada by Heifetz and Lunsky (2018) highlighted that adults with intellectual disabilities and their families reported that a Hospital Passport provides helpful information, improved communication between patients and hospital professionals, and were viewed as user friendly. However, another review of documents including the Hospital Passport by Northway et al. in 2017 identified that variations existed between terminology used to describe Hospital Passports; with significant variations in length and format of the passport, coupled with a poor understanding of how the tool should be used to inform and improve care, thereby limiting their overall effectiveness. Therefore, greater standardisation of the Hospital Passport was recommended across the studies, with further research required to fully evaluate their effectiveness (Bell, 2012; Blair, 2011; Northway et al., 2017). Additionally, few studies have focused on the experiences of using a specific Hospital Passport from a multi-dimensional perspective, including the experiences of adults with intellectual disabilities and are largely absent from the existing research evidence.

The introduction and implementation of the *Regional Hospital Passport* in Northern Ireland arose from a recommendation for the need to improve the hospital experience and health outcomes, for people with intellectual disabilities (Guidelines and Audit Implementation Network, 2010; RQIA, 2014). While all Health and Social Care Trusts had developed and were using a locally developed Hospital Passport, there was a lack of standardisation across all acute hospitals. Subsequently, the *Regional Hospital Passport* was developed and implemented for use across all acute hospital settings in Northern Ireland by the Public Health Agency in May 2017 following a pilot study (Millman and Gamble, 2018). Hardcopies of the *Regional Hospital Passport* were sent to each of the five Health and Social Care Trusts and voluntary organisations for wider distribution. A PDF of the *Regional Hospital Passport* was also available for download from the Public Health Agency’s website. The purpose of this study was to identify the experiences of the use of the *Regional Hospital Passport* from the perspective of the adult with intellectual disabilities, their families and health professionals following this implementation.

## Methods

### Design

A qualitative, descriptive design with a data-driven inductive approach informed the process (Braun and Clarke, 2006). The EQUATOR checklist Consolidated Criteria for Reporting Qualitative research (COREQ) (Tong et al., 2007) was used.

### Recruitment

Participants were recruited via email from across the five Health and Social Care Trusts; voluntary and community sector organisations accessed by adults with intellectual disabilities and families; and professional organisations for nurses, doctors, and professions allied to health in Northern Ireland. Additionally, details of the study were posted on social media sites including Twitter and Facebook. The main inclusion criteria were that participants had used the *Regional Hospital Passport* and were aged 18 years or over and able to provide informed consent.

A total of 29 potential participants contacted the researcher of which 15 were eligible to participate in the study. The eligible participants were 14 females and one male, and included two adults with intellectual disabilities, three family carers, one paid carer and nine registered nurses. On receipt of the study information the paid carer decided not to continue. Despite follow up, one family carer and one health professional, did not progress to an interview. A total of 12 participants provided informed consent and participated in the study (Table 1). Pseudonyms were assigned to provide anonymity and confidentiality was assured.

**Table 1.** Participants’ details.

Participant	Role
Edna	Adult with intellectual disabilities
Linda	Adult with intellectual disabilities
Audrey	Mother to Mark
Tess	Sister to John
Anna	Community Learning Disability Nurse
Catherine	Registered Nurse
Emily	Registered Nurse in Learning Disability
Helen	Learning Disability Liaison Nurse
Irene	Registered Nurse in Outpatients Department
Lucille	Specialist Nurse in Learning Disability
Nicola	Lead Nurse in Learning Disability
Sandra	Learning Disability Nurse in Emergency Department

### Interviews

As the aim of the study was to explore participant experiences, a qualitative inductive interview approach was utilised. To accommodate individual preference, each participant was offered the option of a 1-1 telephone interview or face-to face interview. Four participants requested a face-to-face interview which took place in a location chosen by participants, and eight interviews were conducted by telephone. The use of telephone interviews in qualitative research are rarely employed as it is thought the absence of visual and nonverbal cues may compromise the data. However, although there is a lack of research evidence on data quality, the benefits of accessibility and reduced cost need to be considered (Johnson et al., 2021; Novick, 2008).

An interview schedule developed by the researcher was used to guide each interview. Questions for adults with intellectual disabilities and family carers asked about their experiences of using the *Regional Hospital Passport*, what they liked and disliked about the passport, and what would make the *Regional Hospital Passport* better. The Registered Nurses were also asked about their experiences of using the *Regional Hospital Passport*, what they found useful and the potential barriers to the utilisation of the *Regional Hospital Passport*, and what would they like to see included in best practice guidelines. Probing questions were also asked to encourage participants to extend or elaborate on their responses, although it was notable that the adults with intellectual disabilities gave very succinct answers. The interviews with the researcher lasted an average of 14 minutes for the adults with intellectual disabilities and 28 minutes for family members and Registered Nurses. All interviews were recorded with consent and transcribed verbatim and analysed.

### Data analysis

Transcriptions were thematically analysed to identify key themes. The research team manually analysed the data, thereby developing a close and subjective relationship with the data analysis process (Silverman, 2010). Initial coding of data into categories was performed by the first author and checked for consensus by the second author. The first author then undertook a second level of coding that identified a smaller number of core themes (Caldwell et al., 2011; Saldana, 2009). The proposed themes were then discussed by the research team and consensus agreed.

### Ethics

An ethics application and research protocol were submitted to the Integrated Research Application System. The Proportionate Review Sub-committee of the London-Surrey Borders Research Ethics Committee reviewed the application and approval was granted. All participants received a participant information document by post or as an email attachment. An easy read participant information booklet and consent form which included the use of pictures, colour, and larger font was shared with participants with intellectual disabilities. After receiving written and oral information about the study, participants returned their signed consent forms by post or email.

## Findings

The responses from adults with intellectual disabilities, their families and health professionals regarding their experiences of using the *Regional Hospital Passport* for people with intellectual disabilities are presented in three themes: (i) Usefulness of the *Regional Hospital Passport*; (ii) Facilitators to the use of the *Regional Hospital Passport;* and (iii) Barriers to the use of the *Regional Hospital Passport*. Each theme also had sub-themes, detailed in Table 2.

**Table 2.** Participants’ experiences of using the *Regional Hospital Passport.*

Themes	Sub-themes
Usefulness of the Regional Hospital Passport	Identity acquisition and person-centred care Communication Reasonable adjustments
Facilitators to the use of the Regional Hospital Passport	Content and layout Learning Disability awareness and training Responsibility
Barriers to the use of the Regional Hospital Passport	Reluctance and avoidance to read and use Limited information and resources

### Theme 1: Usefulness of the Regional Hospital Passport

The *Regional Hospital Passport* was viewed by all participants as a useful tool across inpatient admissions and outpatient appointments, and in General Practice and Dental surgeries. It was viewed as a valuable resource by Registered Nurses when supporting adults with intellectual disabilities and their families, as communication pathways were enhanced and key information was readily available, notably when attended the Emergency Department.

#### Identity Acquisition and Person-Centred Care

The information documented within the *Regional Hospital Passport* were considered important and invaluable by the family carers and Registered Nurses and was viewed as a tool to promote person-centred care. For example, preferences for sitting in a chair rather than lying in a bed were documented, which reflected the care required. Tess recalled how her brother John’s and her own experiences with acute hospitals had improved since the *Regional Hospital Passport* was introduced. Tess attributed this positive experience to professionals having a greater understanding of John’s specific needs as they took time to read his information held in the *Regional Hospital Passport*. Additionally, Tess believed health professionals saw John as an individual, with his own identity, despite having additional care and communication needs associated with his intellectual disability. It was evident from Tess’s narrative the benefits of the *Regional Hospital Passport*, stating, *“it has taken a lot of pressure off me”*.

Some Registered Nurses also highlighted how the *Regional Hospital Passport* provided them with a means of identifying that a patient in an outpatient or emergency department had an intellectual disability. It enabled reasonable adjustments to be made, such as being seen as soon as possible, or provided with a quiet place to wait, or longer appointment time, thereby avoiding unnecessary anxiety. This information was particularly useful regarding pre-planned appointments following inpatient care as there was an awareness of the individual’s needs by the Registered Nurses and the adjustments required to their care and support. However, despite key information being readily available and the desire to ensure the care journey through acute services was a more positive experience, one Registered Nurse was aware that sometimes there was an unrealistic expectation that adults with intellectual disabilities should be seen as a priority, which was not always seen as realistic or achievable. Despite this, the benefit of having a *Regional Hospital Passport* was evident across participants.

I felt straightaway that my brother suddenly had an identity … was actually a person, despite his learning difficulties. (Tess, sister to John).

It is an excellent way of finding out about that person and their individual needs … It is simplified, it is not a long-winded document, so you are scanning through it and getting the key information … This is a short concise version of getting the relevant key information. (Helen, Learning Disability Liaison Nurse)

#### Communication

Communication experiences were diverse across the participants with adults with intellectual disabilities reporting that the *Regional Hospital Passport* had a positive effect on their General Practitioner visits and communication interactions, with information sharing improved. On another occasion, when Edna was transported to her local hospital by ambulance, her experience with paramedics improved on presentation of her *Regional Hospital Passport*.

They [*General Practitioner and paramedics*] explained everything in steps for me. Why they were doing what they were doing … They more or less put everything that was happening into easier context or easier wording for me. They explained everything in simple words rather than mumble jumble. (Edna, adult with intellectual disabilities)

The value of the information available within the *Regional Hospital Passport* was further highlighted as a rich information resource by Registered Nurses.

He [*patient’s husband*] is just sick of rewriting the same information over and over and over again. He’s really, really on for the hospital passport, so I know it really does help family members whenever they are completed and filled out properly. (Emily, Registered Nurse in Learning Disability)

Similarly, the experience of a family carer was also positive in relation to professionals utilising her brother’s *Regional Hospital Passport* in the Emergency Department as team communication noticeably improved.

I noticed such a difference … It was from the whole approach from the team … they explained to John who they were, and that’s part of the passport, which was great, and what they were doing and what they were going to do. (Tess, sister to John)

While it was evident that the *Regional Hospital Passport* contributed to improved communication, interactions and information sharing with adults with intellectual disabilities, their families and health professionals, all participants acknowledged that further improvements were required. Recommendation for the inclusion of additional questions related to emergency contacts and numbers and contact details of health professionals involved in the person’s care such as General Practitioners, social workers, speech therapists, specialist nurses were made. Further suggestions by families and Registered Nurses were the inclusion of an up-to-date photograph on the front cover to make it more personal and meaningful to adults with intellectual disabilities.

#### Reasonable Adjustments

The availability of the *Regional Hospital Passport* and the information documented within highlighted the requirement for reasonable adjustments to care and support. They ranged from the adult requiring clearer explanations from health professionals, sourcing specialist equipment, providing a more suitable time to attend an appointment. The need to provide reasonable adjustments such as side rooms, fold out beds or reclining chairs to support families and carers and meet their needs were also identified. Information of the specific needs of adults with intellectual disabilities were also shared across disciplines and professionals such as medical secretaries and consultants, thereby aiming to promote person-centred care and support.

Whilst a Learning Disability Liaison Nurse suggested that some health professionals may not be comfortable with the implementation of reasonable adjustments, other Registered Nurses articulated that there was a sense of fear about them and had subsequently been resolved with the implementation of the *Regional Hospital Passport*. In essence this fear and lack of awareness regarding reasonable adjustments since they are a legal requirement, and a failure to make them is likely to place patients at risk, ultimately presenting a barrier to implementation of the *Regional Hospital Passport*, which in itself is a reasonable adjustment. With ongoing experience, and more knowledge of possible reasonable adjustments, Registered Nurses in acute hospitals concluded they were now better placed to provide person-centred care and support.

We weren’t really sure, legality wise, are we supposed to do this, are we not supposed to do this … so long as there is no risk to the patient the reasonable adjustments are fantastic. It is about thinking outside the box. (Catherine, Registered Nurse)

In the past if someone arrived early, staff were reluctant to give them an earlier appointment but now with the passport they will do this. If anyone challenges this, they are told there is a clinical reason for that person being seen ahead of others. (Irene, Registered Nurse in Outpatients Department)

Further use of the *Regional Hospital Passport* positively developed the experience and confidence of Registered Nurses in the care and support of adults with intellectual disabilities in acute hospitals.

In essence the *Regional Hospital Passport* was viewed as instrumental in providing person-centred care and improving communication by many participants in this study. Adults with intellectual disabilities and family carers, as users of the *Regional Hospital Passport*, reported that information in the passport resulted in increased positive interactions with staff who had taken the time to familiarise themselves with it.

I am a lot more confident going to appointments on my own to know that I have got that passport in my handbag. Before I would have had to drag my brother with me or my friends. (Edna, adult with intellectual disabilities).

### Theme 2: Facilitators to the Use of the Regional Hospital Passport

**Figure 1.**
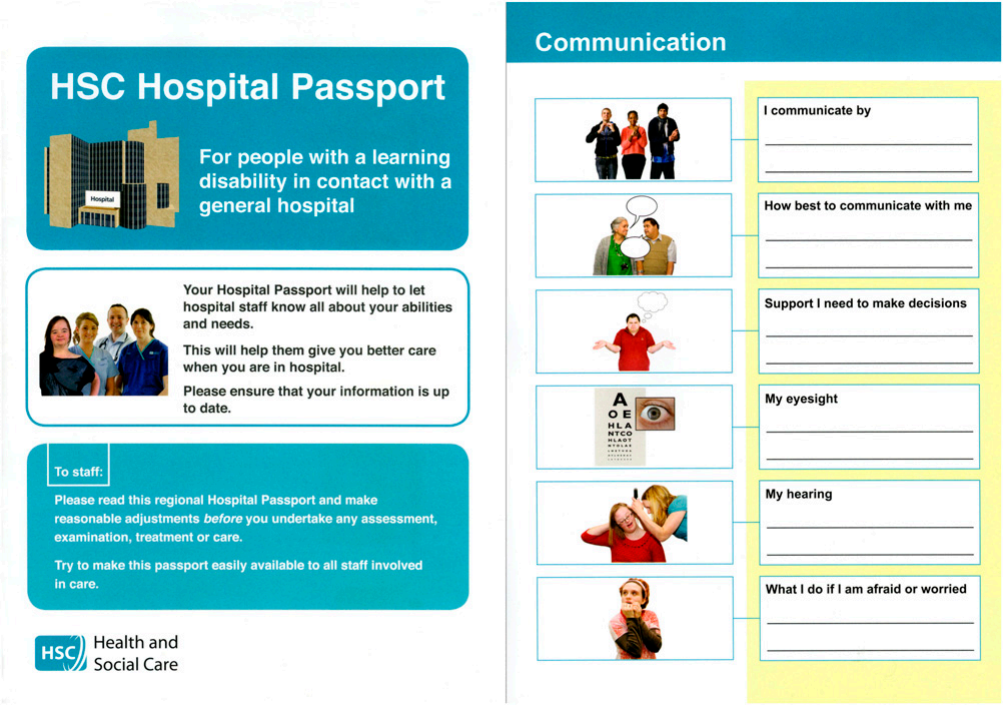
Example from the *Regional Hospital Passport. *

The value of the *Regional Hospital Passport* and ease of use was highlighted overall as an appropriate tool by all participants in this study. However, some recommendations were made as to how it could be improved and developed further. These recommendations were categorised under three themes: content and layout; awareness and training; and responsibility (Figure 1).

#### Content and Layout

There was a mixed reaction to the A4, eight-page, size of the *Regional Hospital Passport* across all groups of participants with Edna commenting it was “*handy enough to put in your handbag*” while Audrey suggested “*it could be smaller*”. However, Emily was equally concerned that “*if it was any smaller it would definitely get lost faster*”. Generally, there was no desire to make the *Regional Hospital Passport* any larger than it currently is. The general consensus across all narratives, was that the short and simple format of the *Regional Hospital Passport* provided sufficient information without the need to “*trawl through notes … to get the key messages*” (Helen, Learning Disability Liaison Nurse).

Although the passport document is titled the ‘*Regional Health and Social Care Hospital Passport*’, it was mainly referred to as a ‘Hospital Passport’. This would suggest that if the title was more generic, such as a Healthcare Passport, adults with intellectual disabilities may be inclined to recognise the value of taking their *Regional Hospital Passport* with them to any healthcare appointment, thereby having it readily available in the event of a referral to an Emergency Department.

Whilst all the questions included in the *Regional Hospital Passport* were relevant to an individual during an inpatient stay, it was recommended to have a shortened version for use in other health care settings, especially in the Emergency Department as the often busy environment may not be conducive to having sufficient time to read the *Regional Hospital Passport* in its current format. There were a variety of responses on how to make information within the *Regional Hospital Passport* more accessible, for example having key information on the first or back page. However, ultimately, this is just a tool and key to its utilisation and success is that it must be tailor made to suit each individual patient irrespective of the healthcare setting they are attending.

It is a bit intimidating for a person with an intellectual disability because they see the words hospital and passport. Hospital means somebody is going to be ill and passport means you are going on holiday. (Audrey, mother to Mark)

Quite often people have gone to the General Practitioner and the General Practitioner has referred them up [*to Emergency Department*] and they just haven’t taken their passport with them. (Sandra, Learning Disability Nurse in Emergency Department)

#### Awareness and Training

Whilst the eight Registered Nurse participants in the study were using and promoting the *Regional Hospital Passport* within their areas of work, there was a general consensus that more awareness and reference to it was critical in supporting health professionals to deliver safe and effective care.

Although some Registered Nurses were delivering awareness and training sessions on intellectual disability which incorporated the *Regional Hospital Passport*, there was a clear position that this training should be mandatory at all staff inductions. Suggestions on the training included incorporation of a case study, shared learning between the multi-disciplinary team and online presentations for staff to access at their own pace. Communication, which included Makaton training, was considered a high training priority. Additionally, the need to include training on the *Regional Hospital Passport* for all healthcare students was recommended.

Initially it should be in everybody’s induction. I know we have nursing and midwifery induction … And then maybe it is something that we should be looking and saying look, every couple of years go for an update … or do short training sessions and update sessions on the ward. (Catherine, Registered Nurse)

While they might never see anybody [*a person with intellectual disability*] in their placement … if they are dealing with somebody, then that [*training*] will be the trigger. (Nicola, Lead Nurse in Learning Disability)

#### Responsibility

In this study, Registered Nurses agreed that the responsibility for the implementation of the *Regional Hospital Passport* in their service be undertaken by all nurses. Some nurses recommended the appointment of a Learning Disability Liaison Nurse within the acute hospital to provide additional support as adults with intellectual disabilities navigate their healthcare journey. The role of the Learning Disability Liaison Nurse was highlighted by a family carer, as a professional well placed to provide additional support and advice regarding the care and support needs of people with intellectual disabilities. A Learning Disability Liaison Nurse stated that while the role is well placed to identify needs, the primary responsibility for completing the *Regional Hospital Passport* and implementing reasonable adjustments, remain the remit of all health professionals rather than one expert, to ensure it was fully utilised.

Some participants identified the need for strategic leadership for the implementation and utilisation of the *Regional Hospital Passport* including specialised intellectual disability and mental health services. The continuing and ongoing responsibility for promoting the *Regional Hospital Passport* was suggested to fit within the remit of intellectual disability services and advocates. There is an appreciation that some hospital wards and departments may have less regular contact with adults with intellectual disabilities. A Lead Nurse from Learning Disability Services emphasised that the *Regional Hospital Passport* still needed to be “*maintained on the agenda*” at strategic level to ensure positive health experiences by adults with intellectual disabilities and their families are a priority of care.

Maybe if it was higher up it would not be a big priority for them to get it implemented … being at ward level it can be spread a lot quicker. It can be managed a lot better at ward level as well … definitely ward level would be a lot more person centred. (Emily, Registered Nurse in Learning Disability).

We have to start putting the onus on all staff within all settings to say, do you know what, this person has a learning disability, do they have a hospital passport, do they need reasonable adjustments for their care, and we need to keep communicating that language among all staff. Not just staff in learning disability teams, not just staff in primary care, not just staff in the acute sector, it is staff throughout the whole of the National Health Service. (Helen, Learning Disability Liaison Nurse)

### Theme 3: Barriers to the Use of the Regional Hospital Passport

#### Reluctance and avoidance to read and use

Irrespective of where the *Regional Hospital Passport* was presented, such as, in the ward setting, the emergency or dental departments, a recurring concern raised by some participants, adults with intellectual disabilities, family carers, and Registered Nurses, was that the *Regional Hospital Passport* was not always read by health professionals due to other competing demands and time constraints.

On two separate occasions when Audrey stayed with her son Mark when he was an inpatient on a ward, even though he had a *Regional Hospital Passport*, she was saddened that staff were still reluctant to make use of it, preferring instead to ask her the same questions. These experiences were also familiar to Anna, a Community Learning Disability Nurse. As a result of hospital staff not always referring to or using the *Regional Hospital Passport*, Anna regularly received phone calls from hospital staff requesting information that was already documented and available in the *Regional Hospital Passport* on admission. Anna was also concerned that families were reluctant to leave their family member alone in hospital in case information was required even when the *Regional Hospital Passport* was available.

On arrival to the Emergency Department, Edna also encountered some negative experiences due to a lack of communication with some health professionals as “*nobody was coming to let me know what was happening*”. Furthermore, Edna highlighted that she was unaware of what happened to her *Regional Hospital Passport* when she arrived at the hospital or who it had been handed over to and it was never returned to her. While she appreciated that “*the staff in Accident and Emergency do not have the time to sit and read all that in the time you are sitting there*”, she was understandably upset as she had to develop a new *Regional Hospital Passport* on discharge. These negative experiences also reinforced that time and possibly lack of effort to read the *Regional Hospital Passport* as another barrier to delivering safe, effective and informed care.

When people see documents, there will be people that will read them, but there will be other people who may not read them. In a busy hospital environment, particularly medics and doctors, they may not read that, and sometimes it’s those particular individuals that probably do need to read it and need to communicate with the person with a learning disability. (Lucille, Specialist Nurse in Learning Disability)

Carers would stay with the person in the ward and are afraid to leave for something to eat, or for a break, in case information is needed. If hospital staff would read the passport this information is already there. (Anna, Community Learning Disability Nurse)

#### Limited information and resources

Not only was it noted that some health professionals did not read the *Regional Hospital Passport*, but there were concerns that some families, carers and health professionals were unaware of its existence. Every effort had been made by the Registered Nurse participants, particularly in outpatient departments and the Emergency Department to promote the *Regional Hospital Passport*. This promotion included posters being displayed and supplies of hard copy *Regional Hospital Passports* being readily available or signposting to an electronic copy.

It was also commented across all participants, that there was insufficient space available to provide more detailed information to some questions with more taken up by pictures and boxes which could be utilised more effectively. The Learning Disability Liaison Nurse was anxious that this limitation could potentially lead to a lack of confidentiality as information was subsequently displayed above the patient’s bed or on a notice board for all to view. The wording of some of the questions and detail provided was highlighted as a concern for a family carer and a Registered Nurse as the way in which some questions were asked did not elucidate the relevant information required. For example, Audrey found the words ‘main carer’ was not reflective of her son’s situation and suggested “*it should just have read ‘who looks after me’’’* whilst Lucille had “*seen some hospital passports where information was scarce*”.

There was an overall sense of frustration by health professionals when an adult with intellectual disabilities was not ‘flagged’ at time of referral. Due to this lack of communication between the community and acute settings there was a lack of processes in place in advance of an appointment, especially notable when an adult with intellectual disabilities was attending outpatient appointments.

The need to ensure the *Regional Hospital Passport* was up to date was another concern raised by some Registered Nurses. Suggestions to negate this included the availability of an App and/or electronic version of the *Regional Hospital Passport* included on the Health and Social Care central databases further retaining its currency. An online version of the *Regional Hospital Passport* would have additional benefits such as less reliance on handwriting, printing and readily available electronic information at hand that was easily accessible.

We haven’t quite got there with the acute sector staff because they have no way of knowing that person has a learning disability until they present actually on the day unless they are previously known to them and they put an alert on them for next appointments. (Helen, Learning Disability Liaison Nurse).

The problem is with the hospital passport, it’s only useful for so long and things need updated regularly and then it’s who is responsible for updating things. (Catherine, Registered Nurse)

## Discussion

The aim of this study was to explore the experiences of the use of the *Regional Hospital Passport* from the perspectives of adults with intellectual disabilities, families and health professionals. Heslop et al. (2013) identified many aspects relating to poor quality of care for people with intellectual disabilities and issues with care pathways, including a lack of reasonable adjustments to help people access healthcare and healthcare information, particularly in relation to accessing non-emergency acute care, a view further supported by Moloney et al.’s (2021) scoping reviews of reasonable adjustments.

The Hospital Passport is considered a useful tool to provide essential information to aid communication with people with intellectual disabilities when attending health services, however, they are often under-utilised in acute hospital settings (Howie et al., 2021). Whilst there are some studies presenting the challenges and experiences of people with intellectual disabilities in acute hospital settings (Drozd et al., 2020; Iacono et al., 2014; McCormick et al., 2020), and some studies reviewing Hospital Passports for people with intellectual disabilities (Heifetz and Lunsky, 2018; Northway et al., 2017), there is an absence of studies exploring a combination of these experiences. All participants in this study championed the value of utilising the *Regional Hospital Passport* and there was a willingness to use it to plan and support the delivery of safe, effective and person-centred care. The findings support the position that the Northern Ireland *Regional Hospital Passport* can improve the experiences of adults with intellectual disabilities and their families when attending acute hospital and other health services. The *Regional Hospital Passport* may also have wider utility as it has the potential to be used with other population groups such as patients with dementia and older adult services.

Previous researchers have also revealed that registered nurses have a need to more fully understand and communicate with people with intellectual disabilities and therefore require a deeper understanding of the reasonable adjustments to support safe and effective care to improve the hospital experience for these patients (Ndengeyingoma and Ruel, 2016; Tuffrey-Wijne et al., 2014). Registered Nurses in this study reported that the *Regional Hospital Passport* assisted them to communicate more effectively with adults with intellectual disabilities and their families, supporting the identification and implementation of reasonable adjustments to enable person-centred care (McCormack and McCance, 2006; Moloney et al, 2021). The use of an up-to-date Hospital Passport for people with intellectual disabilities is advocated by Alexander et al. (2020) to minimise risk in the provision of care and support, notably during the COVID-19 pandemic.

Although the *Regional Hospital Passport* was regarded as a valuable resource, adults with intellectual disabilities, families and Registered Nurses highlighted the need for the current content and format to be reviewed and further developed to increase utility. Areas identified for review extend findings of previous review studies of Hospital Passports, including clearer wording, patient photographic identity, additional space for additional information to be included, such as health status changes (Heifetz and Lunsky, 2018; Northway et al., 2017). Additionally, the ability to access key information, particularly in an emergency, was identified as a necessary development. Therefore, there is scope to develop and include a section with ‘important information’ at the start of the *Regional Hospital Passport*. The title of the *Regional Hospital Passport* also requires further review as reference to ‘hospital’ was identified as limiting the wider utilisation beyond hospitals including General Practitioner and dental practices and community health services.

Howie et al. (2021) suggests that the lack of confidence in supporting people with intellectual disabilities is largely associated with inadequate education. Therefore, Registered Nurses in the acute hospitals often feel underprepared to provide effective care and support. To address this need, intellectual disability awareness and training sessions, including information regarding the use of the *Regional Hospital Passport*, could be developed across health and social care organisations. Previous research has shown that the success of wider implementation of a Hospital Passport requires ‘*buy in*’ from key stakeholders, including acute hospital professionals, families and community practitioners (Heifetz and Lunsky, 2018). This study has also highlighted the additional need for ‘*buy in*’ at a strategic level within the Northern Ireland Health and Social Care Trusts and the appointment of Learning Disability Liaison Nurses to support wider implementation. A Northern Ireland wide communication and disseminations strategy could be developed and implemented to raise awareness, uptake and use of the *Regional Hospital Passport*. All Health and Social Care Trusts could identify a senior person with responsibility for the strategic implementation of the *Regional Hospital Passport* to ensure maximum implementation and utilisation across all areas of Health and Social Care services and integration into their respective Clinical Governance and Patient Safety programmes.

Promoting further access and use of the *Regional Hospital Passport*, through the development and implementation of a Healthcare Passport App was an area identified as necessary. A Healthcare Passport App has the potential to provide easily accessible and up-to-date information for health professionals to access due to the use of smart phone technology, thereby further promoting safe and effective care and support (Alexander et al., 2020). Furthermore, where patients are admitted to hospital unaccompanied by family or carers and visiting not permitted, such as during the COVID-19 pandemic, essential information would be readily available to assist in the planning and delivery of care and support.

## Strengths and limitations

The findings in this study present the views and experiences of adults with intellectual disabilities, families and Registered Nurses from five Health and Social Care Trusts in Northern Ireland. However, the recruitment of adults with intellectual disabilities and families, were less than anticipated. The inclusion criteria for participants to have used the *Regional Hospital Passport* and the impact of the COVID-19 pandemic from mid-March 2020 was identified as two factors effecting participation. The only healthcare professionals involved in this study were Registered Nurses, and whilst they are key to the utilisation of the *Regional Hospital Passport*, nevertheless, it has the potential to be adopted and implemented by all healthcare professionals and acute care staff. The study reports on a variety of settings where the *Regional Hospital Passport* has been used. However, further insight from wider clinical settings, such as primary care and dental services, and some quantitative data, may strengthen the findings.

## Conclusion

These findings support the position that professionals in health and social care services are accountable for ensuring the *Regional Hospital Passport* is promoted and used across health settings in Northern Ireland. Previous research demonstrates that Hospital Passports, enable the making of reasonable adjustments by informing care planning and delivery that improves the care journey for people with intellectual disabilities in healthcare settings. To promote the *Regional Hospital Passport*, a strategic approach is required across key stakeholders. There is therefore a need and an opportunity to develop the use and uptake of the *Regional Hospital Passport* as part of clinical governance and patient safety initiatives that deliver safe, effective and person-centred care and support for people with intellectual disabilities. This is necessary due to the ageing and increasing population of people with intellectual disabilities, many with complex health needs, and there is a need to fully facilitate the uptake and use of the Hospital Passport across all providers of health and social care.

## Supplemental Material

Supplemental Material - Using a hospital passport from the perspective of adults with intellectual disabilities, family carers and health professionals: A qualitative study Supplemental Material for Using a hospital passport from the perspective of adults with intellectual disabilities, family carers and health professionals: A qualitative study by Freda McCormick, Lynne Marsh, Laurence Taggart and Michael Brown in Journal of Intellectual Disabilities
