# Supplementary material for: Using a hospital passport from the perspective of adults with intellectual disabilities, family carers and health professionals: A qualitative study
Source: J Intellect Disabil. 2022 Dec 12;28(1):170–84. doi: 10.1177/17446295221145996 (PMC10916341; doi:10.1177/17446295221145996)
Supplement: Supplemental Material - Using a hospital passport from the perspective of adults with intellectual disabilities, family carers and health professionals: A qualitative study [file sj-pdf-1-jld-10.1177_17446295221145996.pdf]

Consolidated criteria for reporting qualitative studies (COREQ): 32-item checklist

Developed from:
Tong A, Sainsbury P, Craig J. Consolidated criteria for reporting qualitative research (COREQ): a 32-item checklist for interviews and focus groups. *International Journal for Quality in Health Care*. 2007. Volume 19, Number 6: pp. 349 – 357

YOU MUST PROVIDE A RESPONSE FOR ALL ITEMS. ENTER N/A IF NOT APPLICABLE

**No. Item**	**Guide questions/description**	**Reported on Page #**
**Domain 1: Research team and reflexivity**		
Personal Characteristics		
1. Interviewer/facilitator	Which author/s conducted the interview or focus group?<br>Interviews were conducted by FMC	N/A
2. Credentials	What were the researcher's credentials? e.g. PhD, MD<br>FMC – EdD<br>LM – DN<br>LT – PhD<br>MB – PhD	N/A
3. Occupation	What was their occupation at the time of the study?<br>FMC – Research Fellow<br>LM – Senior Lecturer<br>LT – Reader<br>MB – Professor, Director of Graduate Studies	N/A
4. Gender	Was the researcher male or female?<br>Female	N/A
5. Experience and training	What experience or training did the researcher have?<br>FMC – Experience in semi-structured interviewing while pursuing post-graduate studies plus one year of qualitative research experience with older adults with intellectual disabilities.	N/A
Relationship with participants		
6. Relationship established	Was a relationship established prior to study commencement?<br>FMC – one participant had a previously established relationship with the	N/A

No. Item	Guide questions/description	Reported on Page #
	interviewer (colleagues in volunteer training).	
7. Participant knowledge of the interviewer	What did the participants know about the researcher? e.g. personal goals, reasons for doing the research<br>All participants were given an information sheet with details of the research and contact details for FMC and LM.	p.3 Recruitment
8. Interviewer characteristics	What characteristics were reported about the interviewer/facilitator? e.g. bias, assumptions, reasons and interests in the research topic<br>None	N/A
**Domain 2: study design**		
Theoretical framework		
9. Methodological orientation and Theory	What methodological orientation was stated to underpin the study? e.g. grounded theory, discourse analysis, ethnography, phenomenology, content analysis<br>Thematic content analysis	p.5 Data analysis
Participant selection		
10. Sampling	How were participants selected? e.g. purposive, convenience, consecutive, snowball<br>Purposive sampling and snowballing	p.3 Recruitment
11. Method of approach	How were participants approached? e.g. face-to-face, telephone, mail, email<br>Recruitment involved email and distribution of posters.	p.3 Recruitment
12. Sample size	How many participants were in the study?<br>Twelve – two adults with intellectual disabilities, two family carers and eight registered nurses.	p.3 Recruitment
13. Non-participation	How many people refused to participate or dropped out? Reasons?<br>A total of 29 interested people contacted FMC and were asked screening questions to determine if they had used the Regional HSC Hospital Passport. Twelve were excluded as they had not used the	p.3 Recruitment

No. Item	Guide questions/description	Reported on Page #
	Regional HSC Hospital Passport; two made no further contact following initial interest; one did not return the consent form; and two did not progress to an interview.	
Setting		
14. Setting of data collection	Where was the data collected? e.g. home, clinic, workplace<br>The data was collected at the participants' convenience – three in the workplace; one in a hotel lobby; eight by telephone.	p.4 Interviews
15. Presence of non-participants	Was anyone else present besides the participants and researchers?<br>No non-participants were present during data collection.	N/A
16. Description of sample	What are the important characteristics of the sample? e.g. demographic data, date<br>Twelve participants were interviewed from all health and social care Trusts in Northern Ireland. These included two adults with intellectual disabilities, two family carers, and eight registered nurses.	p.3 Recruitment
Data collection		
17. Interview guide	Were questions, prompts, guides provided by the authors? Was it pilot tested?<br>One interview guide was prepared for adults with intellectual disabilities and family carers, and a separate one for registered nurses. It was not pilot tested.	p.4 Interviews
18. Repeat interviews	Were repeat interviews carried out? If yes, how many?<br>No repeat interviews were carried out.	N/A
19. Audio/visual recording	Did the research use audio or visual recording to collect the data?<br>All interviews were audio recorded and transcribed.	p.4 Interviews
20. Field notes	Were field notes made during and/or after the interview or focus group?<br>No field notes were made.	N/A

No. Item	Guide questions/description	Reported on Page #
21. Duration	What was the duration of the interviews or focus group?<br>Each interview lasted an average of 14 minutes for adults with intellectual disabilities and 28 minutes for family carers and registered nurses.	p.4 Interviews
22. Data saturation	Was data saturation discussed?<br>The number of participants, particularly adults with intellectual disabilities and family carers, was less than anticipated. This was contributed to the inclusion criteria for participants to have used the Regional HSC Hospital Passport and also the impact of the COVID-19 pandemic from mid-March 2020.	p.19 Strengths and limitations
23. Transcripts returned	Were transcripts returned to participants for comment and/or correction?<br>No transcripts were returned to participants for comment or correction as they were anonymised at the time of transcription.	N/A
**Domain 3: analysis and findings**		
Data analysis		
24. Number of data coders	How many data coders coded the data?<br>All transcripts were coded by FMC; however, LM read transcripts and provided analytical insights that informed coding.	p.5 Data analysis
25. Description of the coding tree	Did authors provide a description of the coding tree?<br>No description of the coding tree is provided.	N/A
26. Derivation of themes	Were themes identified in advance or derived from the data?<br>Themes were derived from the data.	p.5 Data analysis
27. Software	What software, if applicable, was used to manage the data?<br>Taking into consideration the small-scale nature of the study it was decided to manually analyse the data. An Excel spreadsheet was used.	p.5 Data analysis

No. Item	Guide questions/description	Reported on Page #
28. Participant checking	Did participants provide feedback on the findings?<br>Participant checking was not performed.	N/A
Reporting		
29. Quotations presented	Were participant quotations presented to illustrate the themes/findings? Was each quotation identified? e.g. participant number<br>Participant quotations are provided in the results to illustrate findings. Each quotation is identified by a pseudonym.	p.6 Findings
30. Data and findings consistent	Was there consistency between the data presented and the findings?<br>The examples presented are maximally illustrative of the findings.	p.6 Findings
31. Clarity of major themes	Were major themes clearly presented in the findings?<br>The findings present the major themes which are collated in Table 2.	p.6 Findings
32. Clarity of minor themes	Is there a description of diverse cases or discussion of minor themes?<br>Each major theme also had minor themes.	p.6 Findings

Once you have completed this checklist, please save a copy and upload it as part of your submission. When requested to do so as part of the upload process, please select the file type: *Checklist*. You will NOT be able to proceed with submission unless the checklist has been uploaded. Please DO NOT include this checklist as part of the main manuscript document. It must be uploaded as a separate file.
